# CanID-PCR: a quick and low-cost PCR tool to identify *Candida* species on gDNA directly extracted from positive blood bottles

**DOI:** 10.1128/spectrum.01545-25

**Published:** 2025-11-13

**Authors:** Hassan Badrane, Cornelius J. Clancy, M. Hong Nguyen

**Affiliations:** 1 Division of Infectious Diseases, Department of Medicine, University of Pittsburgh 199716, Pittsburgh, Pennsylvania, USA; 2 V.A. Pittsburgh Healthcare System, Pittsburgh, Pennsylvania, USA; Virginia-Maryland College of Veterinary Medicine, Blacksburg, Virginia, USA

**Keywords:** *Candida *identification, PCR tool

## Abstract

*Candida* bloodstream infection carries high mortality, and antifungal-resistant species, such as *Candida auris* and *C. glabrata*, are increasingly common. Delays in species identification can adversely affect patient outcomes. Conventional workflows by clinical microbiology laboratories require ~24 hours for *Candida* speciation to allow isolate growth on agar plates. We developed CanID-PCR, a simple, inexpensive PCR-based tool for rapid *Candida* identification directly from positive blood culture bottles. The assay targets the *ACT1* gene, which contains a variable-length intron enabling discrimination among nine common *Candida* species. CanID-PCR was optimized for genomic DNA extracted directly from positive blood culture bottles and tested on 64 patient samples. Overall, species identification matched the clinical microbiology laboratory with 99% of concordance. One limitation was the inability to distinguish *C. parapsilosis* from *C. metapsilosis*, which are highly similar members of the *C. parapsilosis* complex. The unique discrepancy involved a failure to detect *C. tropicalis* in a mixed infection with *C. glabrata*. Among a total of seven samples with two species, one sample had a second species: *C. fabianii* (*Cyberlindnera fabianii*), detected only by CanID-PCR, but was missed by the clinical laboratory. These findings demonstrate that CanID-PCR can reveal polymicrobial *Candida* infections often overlooked when only a few selected colonies are analyzed. CanID-PCR provides rapid, accurate *Candida* speciation without the need for additional culture, requiring only standard laboratory equipment. This low-cost, straightforward approach is suitable for research laboratories, resource-limited clinical settings, or scenarios where timely species-level identification is critical.

**IMPORTANCE:**

Identifying species in *Candida* infections is of utmost importance to clinicians and researchers alike. Here, we describe CanID-PCR: a quick and low-cost PCR tool to identify *Candida* species on genomic DNA directly extracted from positive blood cultures. This technique uses basic laboratory equipment, and there is no need for high investment in special equipment. The tool allows identification of the nine most prevalent species in candidiasis. The technique is simple and can be used without the need for an additional culture to isolate the cells. It is noteworthy to mention that this tool can detect polymicrobial infections caused by more than one *Candida* species.

## INTRODUCTION

*Candida* is the most common cause of nosocomial fungal bloodstream infection (BSI), a condition associated with high morbidity and mortality. The most common causes of *Candida* BSI are *C. albicans*, *Nakaseomyces glabratus* (previously known as *C. glabrata*), *C. parapsilosis*, *C. tropicalis*, and *Pichia kudriavzevii* (previously known as *C. krusei*) (1), although emerging species such as *C. auris* (*Candidozyma auris*) are increasingly reported (2). Rapid and accurate *Candida* species identification is important in clinical practice, as it informs the likely resistance profiles and guides timely antifungal therapy (3, 4). It is also important for epidemiological and bench research purposes.

Various approaches, including phenotypic, proteomic, and molecular methods, have been developed to speciate *Candida* isolates in clinical laboratories (5). Conventional phenotypic/biochemical methods are laborious, time consuming, and occasionally inaccurate. Molecular techniques have been applied to a limited number of *Candida* species and frequently require multiple targets, which can make them complex and impractical. Finally, commercial instruments (like the widely used matrix-assisted laser desorption ionization–time-of-flight mass spectrometry [MALDI-TOF MS]) provide rapid and generally accurate results but require substantial capital investment (6). Most methods rely on the recovery of the fungal isolate through culture, which usually adds ~24 hours to the identification timeline. Recently, molecular techniques such as multiplex PCR platforms (e.g., Biofire or Cobas eplex) have been introduced for direct identification from positive blood culture samples (7). While effective, these systems require costly instruments and reagents, as well as integration with existing laboratory workflow. Given the lower prevalence of fungal BSIs compared to bacterial BSIs, such investments may not be cost-effective or practical in all settings.

In this study, we describe a PCR-based tool for the rapid identification of *Candida* species directly from genomic DNA (gDNA) extracted from positive blood bottles. This approach bypasses the need for an additional 24-hour culture step, requires only standard laboratory equipment, and can provide species-level identification in approximately 8 hours. Unlike many other methods, this tool can also detect polymicrobial *Candida* bloodstream infections in which more than one species is present, a feature that may be missed because conventional laboratory workflows typically analyze only a few selected colonies. While not intended to replace high-throughput clinical platforms, this method offers a practical alternative for research laboratories, resource-limited settings, or in situations where rapid species identification is needed but investment in expensive equipment is unfeasible.

## MATERIALS AND METHODS

### Strains and growth conditions

The sources of *C. albicans*, *C. glabrata*, *C. tropicalis*, *C. krusei*, *C. tropicalis*, *C. parapsilosis*, *C. lusitaniae* (also known as *Clavispora lusitaniae)*, *C. auris*, and *C. dubliniensis* are listed in Table 1. For clarity, both the clinical name and the currently accepted taxonomic name are provided in the introduction and Table 1. Clinical names (e.g., *Candida* species) are used throughout the manuscript to maintain consistency with standard clinical practice while acknowledging updated taxonomy. The identification of *C. fabianii* and other isolates was performed using MALDI-TOF MS (Bruker Biotyper, library version 10.0, Bruker Daltonics, Billerica, MA, USA). *C. fabianii* was recovered from a patient’s blood culture in our research laboratory, where species identification was confirmed by MALDI-TOF MS. For all control isolates, including *C. fabianii*, species confirmation was performed by Sanger sequencing of the internal transcribed spacer (ITS) region and the D1/D2 domain of the 28S ribosomal DNA. Strains were grown in yeast-peptone-dextrose, Synthetic Defined, or CHROMAgar Candida (CHRCA) media at 30°C or 37°C.

**TABLE 1:** *Candida* reference isolates used in this study

Species (clinical name)	Taxonomic name	Strain	Reference	ACT1 band size (bp)
*Candida orthopsilosis*	Same	CDC317	8	519
*Candida auris*	*Candidozyma auris*	B8441 (AR-0387)	9	589
*Candida lusitaniae*	*Clavispora lusitaniae*	ATCC 66035	10	644
*Candida parapsilosis/metapsilosis*	Same	ATCC 22019	11	733
*Candida dubliniensis*	Same	ATCC MYA-646	12	767
*Candida albicans*	Same	SC5314	13	793
*Candida tropicalis*	Same	ATCC 20336	14	824
*Candida fabianii*	*Cyberlindnera fabianii*	62862-CF60A	Present study	861
*Candida glabrata*	*Nakaseomyces glabratus*	CBS138	15	1,012
*Candida krusei*	*Pichia kudriavzevii*	ATCC 6258	16	1,144

### gDNA extraction directly from positive blood bottles

About 10 mL of broth from positive blood culture bottles was treated twice with 2× volume of alkali solution (0.1 M sodium citrate and 1 M sodium hydroxide) (17) for 10 min with rotation at 15 rpm. The sample was then centrifuged at 3,155 *× g* for 5 min. Pellets were resuspended in 1 mL of sterile nuclease-free water, centrifuged again, and finally resuspended in 600 µL extraction buffer (CLS-Y) from FastDNA Kit (MPBIO, San Diego, CA, USA). Cell lysis was performed in the provided tubes with beads using a beadbeater (Biospec Products, Bartlesville, OK, USA) for 2 × 60 s at maximum speed with a 60-second interval on ice between runs. DNA extraction was then carried out per the manufacturers’ instructions. Eluted gDNA (200 µL) was further purified using Wizard SV Gel and PCR clean-up System (Promega, Madison, WI, USA) and finally resuspended in 40 µL nuclease-free water.

### Fingerprinting using random amplified polymorphic DNA (RAPD)

Ten-mer RAPD primers were designed for DNA fingerprinting of *Candida* isolates (Table 2). RAPD was prepared as a 25 µL total volume containing 1 µL of gDNA, 2 µL of 5 µM RAPD primer, 0.5 µL of 10 mM dNTP mix, 2.5 µL of 10× Taq buffer, 0.5 µL enzyme (5 U/µL), and nuclease-free molecular biology grade water adjusted to a total volume of 25 µL. Thermocycling was performed on an MJ Research Diad thermocycler (Biorad, Hercules, CA, USA) using the following program: (i) an initial denaturation at 94°C for 1 min; (ii) 35 cycles of 94°C for 15 s, primer-specific annealing temperature 32°C–34°C for 30 s (Table 2), and 68°C for 40 s; and (iii) a final extension at 68°C for 10 min. PCR products were separated on a 1% agarose gel in 1× Tris-acetate-EDTA for 1.5 hours at 80 V.

**TABLE 2:** Sequence and melting temperature (Tm) of primers used for the RAPD and CanID-PCR

Oligo name	Sequence	Tm	Purpose
ACT-CPAR-F2	CAAAATGGACGGTGGTATGTATATTTA	56°C	Forward cocktail
ACT-CLUS-F2	GGATGTGTAGGGTGCTGTTT		
ACT-CAUR-F2	TTGCTTCGCCTGGCCAATCT		
ACT-CorP-F2	TGTCGAGAGAGAATGATGACGA		
ACT-CALB-F	CAAAATGGACGGTGGTATGTTT		
ACT-CDUB-F	AAAAATGGACGGTGGTATGTTT		
ACT-CTRO-F	AAAAATGGACGGTGGTATGTTT		
ACT-CFAB-F	CAAAATGGACGGAGGTACGTAT		
ACT-CKRU-F	AACATGAGTACTAACATTGATTCCCT		
ACT-CGLA-F	TAACAATGGATTCTGGTATGTTCGAC		
ACT-CAN1-REV	ATACCTTGATGTCTTGGTCTACC		Reverse cocktail
ACT-CAN2-REV	ATACCTTGGTGTCTTGGTCTACC		
ACT-CAN3-REV	ATACCCTGGTGTCTTGGTCTACC		
OPAW-17	TGCTGCTGCC	34°C	Random primers
OPBC-12	CCTCCACCAG	34°C	
OPM-13	GGTGGTCAAG	33°C	
OPO-03	CTGTTGCTAC	32°C	

### *ACT1* gene intron PCR for species identification

The *ACT1* gene intron varies in size among *Candida* spp., enabling species discrimination (Table 1; Fig. S1). Ten species-specific forward primers and three conserved reverse primers were pooled into equimolar cocktails (5 µM each for the forward and 7 µM each for the reverse; Table 2).

PCR was performed using 5PRIME Hotmaster Taq DNA polymerase (QuantaBio, Beverly, MA, USA), in a final reaction volume of 25 µL, consisting of 1 µL gDNA, 1 µL each of the forward and reverse primer cocktails, 0.5 µL of 10 mM dNTP mix, 2.5 µL of 10× Taq buffer, 0.5 µL enzyme (5 U/µL), and nuclease-free molecular biology grade water added to reach a total volume of 25 µL. Cycling conditions (MJ Research Diad thermocycler, Biorad, Hercules, CA, USA) using the following program: (i) an initial denaturation at 94°C for 1 min; (ii) nine cycles of 94°C for 15 s, 56°C for 30 s, and 68°C for 40 s; (iii) 32 cycles of 94°C for 10 s, 56°C for 15 s, and 68°C for 35 s; and (vi) a final extension at 68°C for 10 min. Alternatively, PCR was performed using PFU Ultra II Fusion HS DNA polymerase (Agilent Technologies, Cedar Creek, TX, USA) or CesiumTaq Polymerase (DNA Polymerase Technology, Saint Louis, MO, USA) following the manufacturer’s recommendations. PCR products were purified using Wizard SV Gel and PCR clean-up System (Promega, Madison, WI, USA). Purified or crude PCR products were run on a 2% agarose gel in 1× Tris-borate-EDTA for 4 hours at 90 V.

### Alternative protocol using commercial kits

In the latter part of the project, we used MolYsis Basic5 kit (Molzym, Bremen, Germany) for host DNA depletion and microbial enrichment. gDNA was extracted using ZymoBIOMICS DNA Miniprep Kit (Zymo Research, Irvine, CA, USA). PCR amplification was performed using repliQa HiFi ToughMix (Quantabio, Beverly, MA, USA) using the following cycling conditions: (i) an initial denaturation at 98°C for 30 s; (ii) 8 cycles of 98°C for 10 s, 55°C for 20 s, and 68°C for 15 s; (iii) 32 cycles of 98°C for 10 s, 56°C for 15 s, and 68°C for 10 s; and (vi) a final extension at 68°C for 30 s. This alternative protocol demonstrated higher tolerance to PCR inhibitors and reduced hands-on time while maintaining performance for all samples, including those that failed with the manual method.

## RESULTS

### Primer design and RAPD testing on *Candida* colonies from positive blood culture bottles

**Fig 1:**
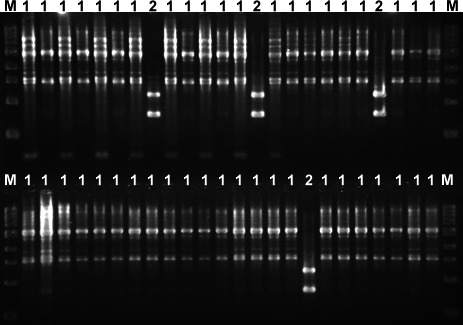
Agarose gel electrophoresis of RAPD PCR products. These RAPD profiles were obtained using primer OPAW-17 on gDNA extracted from clones (usually 96 clones and only 48 shown) isolated from a *Candida* positive blood bottle (this gel corresponds to sample S1 shown on Fig. 3). The gel shows two RAPD profiles 1 and 2 (shown on top of each lane), which will be further identified as *C. parapsilosis* and *C. fabianii*, respectively. M is NEB 1 kb DNA ladder.

We initially aimed to detect polyclonal (mixed strains) *Candida* infections directly from positive blood bottles. RAPD PCR, previously shown to distinguish between Candida strains within the same species (18–23), was applied to colonies subcultured from four positive blood cultures identified by our clinical laboratory as single-species infections (*C. parapsilosis*, *C. glabrata*, or *C. albicans*). We designed a dozen RAPD primers, of which the best four performing primers were selected after optimization. To validate these four RAPD primers, 96 colonies were subcultured onto Sabouraud dextrose agar (SDA) plates from each of the four positive blood cultures and fingerprinted using RAPD. In three samples, the RAPD profiles were indistinguishable. In the fourth sample, two distinct RAPD profiles were observed on agarose gel (Fig. 1), despite identical colony morphologies on SDA and CHRCA plates. Sanger sequencing of the amplified *ITS3* region of the two strains with distinct RAPD profiles confirmed the presence of two distinct *Candida* species in the same blood culture bottle: *C. fabianii* and *C. parapsilosis*. Thus, RAPD PCR detected a polymicrobial *Candida* bloodstream infection that was missed by the clinical microbiology laboratory.

Based on these findings, we designed a PCR assay to identify the nine most common *Candida* species: *C. albicans*, *C. glabrata*, *C. parapsilosis/metapsilosis*, *C. orthopsilosis*, *C. tropicalis*, *C. dubliniensis*, *C. auris*, *C. lusitaniae*, and *C. krusei*, and we included *C. fabianii*, as well. The housekeeping gene *ACT1*, which contains a size-variable intron and a highly conserved downstream region, was chosen as the PCR target. Species-specific forward primers were designed in the variable region just upstream of the ATG start codon, and three conserved reverse primers were designed in a very conserved region downstream of the intron. Thus, the PCR yields species-distinguishable band sizes (except *C. parapsilosis/metapsilosis*, which share high genetic similarity and the same PCR size). Band sizes for each species are summarized in Table 1.

### Development of reference markers

**Fig 2:**
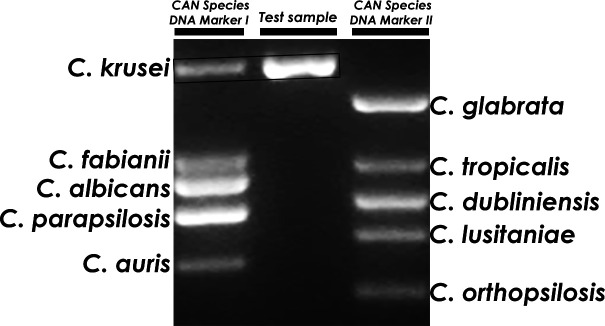
Agarose gel electrophoresis showing CanID-PCR principle. The sample’s PCR product is migrated between two lanes each having a mixture of band size markers for five *Candida* reference species, i.e., Candida species DNA Marker I or II; CAN I or CAN II, respectively (see text for more details). The sample will match one or more band sizes; for instance, the sample shown matched *C. krusei*.

To construct reference markers, *ACT1* PCR products from the 10 *Candida* references were purified and combined into two size markers: CAN Species DNA Marker I (CAN I: *C. krusei*, *C. fabianii*, *C. albicans*, *C. parapsilosis/metapsilosis*, and *C. auris*) and CAN Species DNA Marker II (CAN II: *C. glabrata*, *C. tropicalis*, *C. dublinensis*, *C. lusitaniae*, and *C. orthopsilosis*). Each marker has the bands intercalated by 57–283 bp. Test sample PCR products were run alongside CAN I and CAN II to identify species based on the matching band size (Fig. 2).

### PCR amplification directly from positive blood cultures

**Fig 3:**
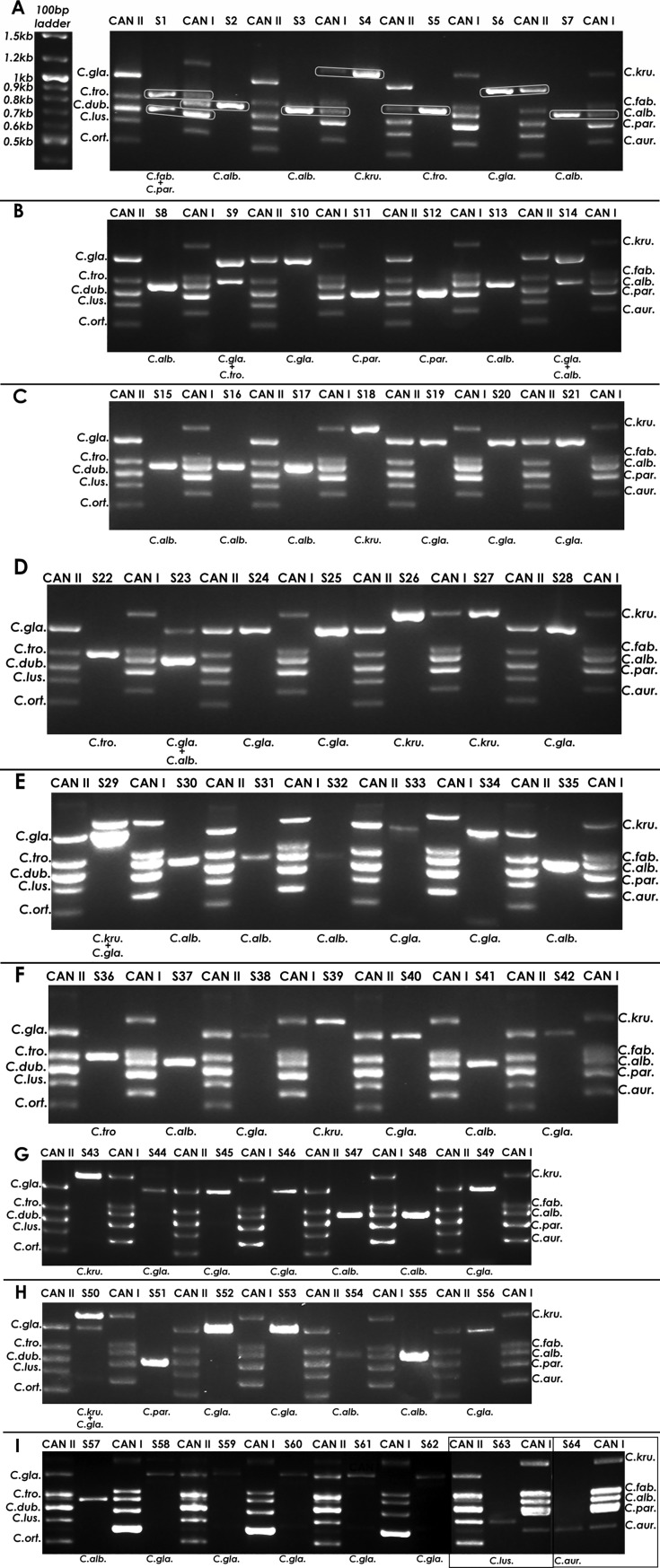
CanID-PCR results for clinical *Candida* positive blood-bottle samples. Agarose gel electrophoresis of CanID-PCR products for 64 (**A–I**) *Candida* samples run along CAN I and CAN II species DNA markers. On panel **A**, a NEB 100 bp ladder is shown for reference, and gray boxes indicate the species band matching for identification. All panels show a single gel, except panel I, which has two small gels (framed with black rectangles) attached to the main gel. The species was successfully identified on all 64 samples and indicated at the bottom of each sample’s lane. C. alb.: *C. albicans*; C. kru.: *Pichia kudriavzevii*; C. fab.: *Cyberlindnera fabianii*; C. dub.: *C. dublinensis*; C. aur.: *Candidozyma auris*; C. gla.: *Nakaseomyces glabratus*; C. tro.: *C. tropicalis*; C. par.: *C. parapsilosis/metapsilosis*; C. lus.: *Clavispora lusitaniae*; C. ort.: *C. ortho*psilosis.

Next, we applied our assay to test 64 positive blood culture samples from 64 unique patients, including the four used for RAPD testing (Fig. 3). Fifty-eight positive blood cultures were caused by a single *Candida* spp. The most common species were *C. glabrata* (*n* = 24), *C. albicans* (*n* = 19), and *C. krusei* (*n* = 6), followed by *C. tropicalis* (*n* = 3), *C. parapsilosis* (*n* = 3), and one each of *C. lusitaniae*, *C. metapsilosis*, and *C. auris* (Table 3). Six samples contained two species, all including *C. glabrata*; the co-infecting species were *C. albicans* (*n* = 2), *C. krusei* (*n* = 2), and *C. tropicalis* (*n* = 2; Table 3). Prior to DNA extraction, as part of our internal control, we sub-cultured the blood culture broth onto CHRCA and SDA plates; recovered species matched the clinical laboratory results in 63/64 bottles. In one bottle with dual *C. glabrata* and *C. tropicalis* identified by the clinical laboratory, CHRCA identified only *C. glabrata*, while *C. tropicalis* could not be isolated.

**TABLE 3:** Comparison of *Candida* species identification methods for positive blood culture samples^*d*^

MALDI-TOF MS identification	CHROMagar *Candida*^*a*^	CanID-PCR
*C. glabrata* (*n* = 24)	100% (24)	100% (24)
*C. albicans* (*n* = 19)	100% (19)	100% (19)
*C. krusei* (*n* = 6)	100% (6)	100% (6)
*C. tropicalis* (*n* = 3)	100% (3)	100% (3)
*C. parapsilosis* (*n* = 3)	100% (3)	100% (3)^*b*^
*C. metapsilosis* (*n* = 1)	0% (0)	0% (0)
*C. auris* (*n* = 1)	100% (1)	100% (1)
*C. lusitaniae* (*n* = 1)	100% (1)	100% (1)
*C. glabrata* and *C. albicans* (*n* = 2)	100% (2)	100% (2)
*C. glabrata* and *C. krusei* (*n* = 2)	100% (2)	100% (2)
*C. glabrata* and *C. tropicalis* (*n* = 2)	50% (1)^*c*^	50% (1)^*c*^

^*a*^ Please note that CHROMagar *Candida* provides a presumptive identification. A result was considered a match when it agreed with the MALDI-TOF MS speciation.

^*b*^ One sample grew *C. fabianii* as well.

^*c*^ Only *C. glabrata* was recovered from the blood culture bottle; *C. tropicalis* was not detected, likely due to poor growth in the anaerobic bottle.

^*d*^ Percentages indicate agreement with the clinical microbiology laboratory (MALDI-TOF MS); numbers in parentheses show the number of matching samples.

PCR amplification was successful in all samples. On gel electrophoresis, a single band was observed in 58 samples, and two bands in six samples. Overall, CanID-PCR identified the same species as the clinical laboratory in 63/64 samples. In one sample, CanID-PCR detected an additional species, *C. fabianii*, missed by MALDI-TOF (sample S1; Fig. 3A). In another sample (S28; Fig. 3D), CanID-PCR identified only *C. glabrata*, while the microbiology lab reported both *C. glabrata* and *C. tropicalis*. Repeated PCR and subculture in CHRCA of this sample confirmed only *C. glabrata*, suggesting that *C. tropicalis* did not grow in the anaerobic bottle tested (Fig. S2).

CanID-PCR cannot distinguish between the genetically very closely related *C. metapsilosis* and *C. parapsilosis* (24), and by default, matching bands were attributed to *C. parapsilosis*. In total, there were seven samples (11%) with two species identified by either the clinical lab or PCR, and among these, one had a species missed by the clinical lab and one had a species missed by CanID-PCR.

## DISCUSSION

Our study demonstrates that CanID-PCR analysis directly from positive blood culture bottles provides accurate identification of the nine most common *Candida* species causing human infections. This approach is simple, rapid, and inexpensive in settings where MALDI-TOF MS instrumentation is not available or feasible due to high cost. We emphasize that our method is not intended to replace MALDI-TOF in well-equipped clinical laboratories but rather offers an alternative for resource-limited environments. MALDI-TOF MS remains the gold standard in many laboratories, as it is rapid and inexpensive once a pure culture is available (USD 1–2 per isolate). However, it requires a substantial capital investment (~USD 200–300kK plus the cost of annual service contracts) and, in most workflows, an additional 16–24 hours for isolate growth. Direct-from-blood MALDI-TOF protocols can shorten turnaround time to 2–4 hours, but these require in-house optimization, are not FDA-cleared, and are not widely implemented. By contrast, CanID-PCR uses standard molecular biology equipment with consumables costing approximately USD 5–8 per test and provides species-level identification within 8 hours of a positive blood culture. While the workflow involves multiple steps and requires trained personnel, it avoids the need for high-cost instrumentation, making it feasible in many academic and research settings worldwide.

We successfully applied CanID-PCR to 64 clinical blood culture samples, identifying the same species as the clinical microbiology laboratory with 99% match. Notably, our tool detected a second species, *C. fabianii*, in one sample (S1) that was missed by the microbiology lab, highlighting the potential of molecular methods to detect polymicrobial infections that may go unnoticed using conventional approaches. In another sample (S28), CanID-PCR identified only *C. glabrata*, consistent with our subculture results, suggesting that *C. tropicalis* may not grow well in anaerobic blood culture bottles (25). These findings underscore the importance of considering growth conditions when interpreting discrepancies between molecular and culture-based methods.

Polymicrobial candidemia was observed in 11% of patients in our cohort, higher than the ~5% rate reported in previous studies (26, 27). Detecting multiple *Candida* species is clinically relevant, as co-infections may involve species with differing antifungal susceptibility profiles. For example, a poly-*Candida* infection including an azole-resistant and an azole-susceptible species could significantly impact treatment decisions. While our tool focuses on species identification rather than antifungal susceptibility, it provides valuable supplementary information that can guide empirical therapy and inform laboratory workflow decisions.

Our primers were designed using the *ACT1* gene intron, with forward primers specific to each species and conserved reverse primers shared across species. Although we did not test other non-targeted organisms that may be encountered in blood culture (e.g., *C. brasiliensis*, *C. nivariensis*, *Cryptococcus neoformans*, and *Meyerozyma guilliermondii*) due to a lack of availability, sequence analysis indicates that our primers are unlikely to amplify these non-target organisms. For instance, *C. brasiliensis* contains multiple ACT1 introns and divergent sequences in the primer binding regions, which will most likely prevent PCR amplification.

Nevertheless, we further showed that gDNA extracted using the manual or the kits protocols was successfully amplified in all samples, although the later approach was less time consuming and more consistent. Overall, CanID-PCR analysis accurately identified the *Candida* species identified by the clinical microbiology lab using MALDI-TOF MS in 63 of 64 samples. It is not optimized yet to distinguish between *C. parapsilosis* and *C. metapsilosis* because of their very close genetic relatedness (24). Indeed, *C. metapsilosis* belongs to the *C. parapsilosis* complex and is phenotypically undistinguishable from other members of this complex and is less common and less virulent than *C. parapsilosis* (28). In one discrepant sample (S28), CanID-PCR detected only *C. glabrata*, while the clinical laboratory identified both *C. glabrata* and *C. tropicalis*. Our follow-up experiments indicated that *C. tropicalis* was not recovered from the anaerobic blood culture bottle used for PCR. This observation aligns with prior studies showing that *C. tropicalis* exhibits reduced growth under anaerobic conditions, whereas *C. glabrata* can grow under both aerobic and anaerobic conditions (25). Therefore, the absence of *C. tropicalis* in our PCR assay likely reflects the growth limitations of this species in anaerobic bottles rather than a failure of the assay itself. Our second important finding is that 11% of our patients with candidemia were infected with two different species. Polymicrobial candidemia has been reported to occur in ~5% of patients (26, 27), but this rate might be underestimated because of the current workflow of the clinical microbiology lab, where only morphologically different clones were selected for susceptibility testing. In this regard, our molecular tool was able to detect a second species, *C. fabianii*, from the blood culture bottle S1.

In conclusion, CanID-PCR provides rapid, accurate species identification directly from positive blood culture bottles, including detection of polymicrobial infections. The method is especially valuable in laboratories without access to MALDI-TOF MS or where rapid molecular detection is desired. By delivering species-level identification within hours and without major capital investment, this tool can complement existing workflows and support both clinical decision-making and epidemiological surveillance of candidemia.

## SUPPLEMENTAL MATERIAL

Supplemental figures spectrum.01545-25-s0001.docx Figures S1 and S2.

ASM does not own the copyrights to Supplemental Material that may be linked to, or accessed through, an article. The authors have granted ASM a non-exclusive, world-wide license to publish the Supplemental Material files. Please contact the corresponding author directly for reuse.
