# Supplementary material for: CanID-PCR: a quick and low-cost PCR tool to identify Candida species on gDNA directly extracted from positive blood bottles
Source: Microbiol Spectr. 2025 Nov 13;14(1):e01545-25. doi: 10.1128/spectrum.01545-25 (PMC12772404; doi:10.1128/spectrum.01545-25)
Supplement: Supplemental figures — Figures S1 and S2. [file spectrum.01545-25-s0001.docx]

**SUPPLEMENTAL MATERIAL**

**CanID-PCR: A quick and low-cost PCR tool to identify *Candida* species on gDNA directly extracted from positive blood bottles**

Hassan Badrane, Cornelius J. Clancy and M. Hong Nguyen


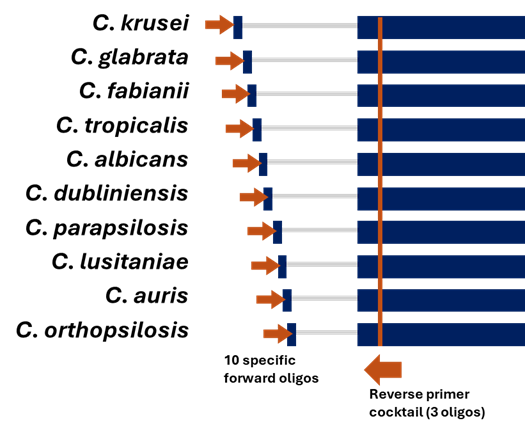


**FIG 1S** Schematic representation of the ACT1 gene organization around the intron across the ten species analyzed in this study. Intron lengths differ between species, providing a basis for differentiation. Arrows indicate primer binding sites: the forward primers cocktail (10 species-specific oligonucleotides) upstream of the intron near the ATG start codon, and the reverse primers cocktail (3 oligonucleotides) targets a very conserved downstream sequence. Primer sequences are provided in Table 2.

**
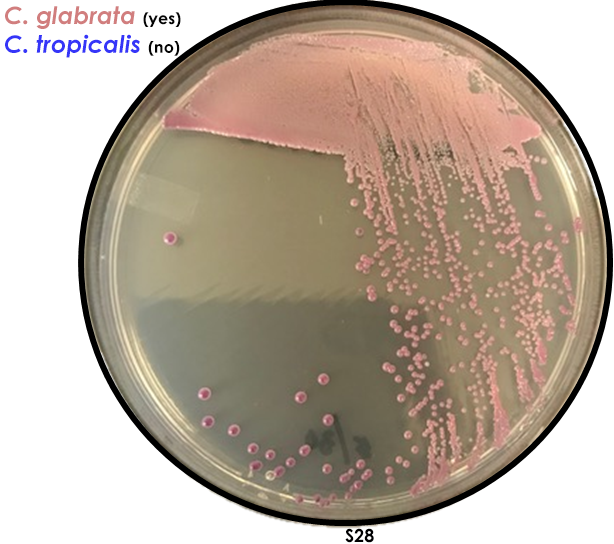
**

**FIG 2S** CHROMagar^TM^ Candida growth plate of sample S28. An aliquot from blood-bottle of sample S28 was re-streaked on CHRCA plate and grown for 48 hours at 30°C. Only *C. glabrata* could be identified, *C. tropicalis*, which produces distinctive metallic blue colored colonies, is not detected. This result concords with CanID-PCR.
